# The New Theology and the New Medicine

**Published:** 1896-05-09

**Authors:** 


### The New Theology and the New Medicine.

Between the body and the soul of man there is a close affinity. For all save the materialist a human being is something more than a curiously agitated mass of bone and muscle. The outer shrine which meets the eye has an inward and spiritual counterpart. It has been for centuries the problem of the metaphysician to discover the nature of the link which unites the two, to throw a bridge between the mind that thinks and the matter in which it is apparently enclosed. If such a connection there be, it follows that there is also an analogy between the healing of the bodies and the souls of men, between theology and medicine. The diagnosis of the doctor not seldom points to evils arising from latent mental or spiritual trouble, whilst the minister is aware that there are cases more suitably dealt with by the physician than by the divine. Christianity, recognising this, has ever taken thought for the bodies of men, and its great Founder did not deem physical necessities outside His province. He multiplied the products of the earth to feed the hungry, and did battle with disease and death. The physician, then, who never warned against the consequences of immorality, or spoke a word of spiritual counsel in season, or the minister who refused to alleviate the bodily wants of men, would surely leave a deficiency in his practice. Each profession finds a corollary in the other, and cannot afford to neglect this secondary province. Here there is a marked difference between Christianity and other religions of ancient times. An examination of the remains of the cities of antiquity reveals the fact that earlier civilisations made little or no provision for the bodies of men. There are relics in abundance of the amphitheatre, the temple, and the forum, but none of any buildings devoted to the care of men's physical wants, no hospital or asylum. To afford a parallel between modern cities and those of the past, we should have to imagine the maniac, the crippled, and the distempered thrust forth upon the public streets. The truth of this analogy between the physical and the spiritual requirements of man is increasingly being recognised. Theologians in the past have been over-ready to discount the value of medical discoveries. To-day, both in theology and medicine, old and narrow ideas are being largely modified, and it will be interesting briefly to trace the development which has taken place with almost even pace in theological and medical theories. And first as to bodily ills. Old cures were harsh and severe. A patient was likely to "suffer much at the hands of many physicians." It is only those of an inquiring turn of mind who can form any conception of the extraordinary curative means adopted by physicians in the past, and, it must be added, the amount of suffering inflicted by them over and above what Nature intended. In times when superstition entered largely into received conceptions on almost every subject, physicians acted upon the belief that diseases were due to the presence of evil spirits in the body. The body must be made as undesirable a tenement as possible; if it was made sufficiently untenable, the theory was that the evil spirit would depart and leave the sufferer in peace.

Our lot is cast in happier and more enlightened times. Such methods of treatment linger on now only in Eastern countries, or amongst the "medicine men" of savage tribes. It is the aim of a more enlightened science to give Nature fair play. The doctor's endeavour is all to aid Nature in her beneficent and restorative work. Thus cures are more frequent, life is prolonged, and the patient suffers less pain than formerly. Now it is interesting to notice that very similar and changed methods of treatment have been adopted by the minister in dealing with the souls of men. As in medicine, so in theology, cures by violence have been abandoned. We no longer see attempts made to extend the borders of Christendom at the point of the sword. The Inquisition tortured the heretic in order to save him from ultimate perdition. In the history of our own country there is no more melancholy reading than those pages which record the endeavours made to inculcate religious beliefs by means of force. We are wiser now, and burning bodies no longer seems a desirable way of saving souls. Legal penalties and political disabilities are no longer used as a means to spread gospel truths. Like the doctor, the minister has adopted a milder form of treatment. He preaches love rather than fear. And yet, be it observed, those who endeavoured to cure either body or soul by violence were often good men and actuated by the best of motives. Their besetting sin was ignorance. When the use of the thumb-screw was given up on earth, torture was frequently held in reserve hereafter. Compulsion was only deferred. Now, save with a few of extreme puritanical sentiments, all is changed. It is the case of the physician and his patient over again. In neither instance is human nature to be tortured out of its natural existence. To enhance the glories of the future it is no longer thought essential to blacken the present. The gloom of the Sabbath-day is being dispelled, and the Legislature has recently decreed the opening of our museums and public galleries upon the day of rest. Sunshine is not to be blotted out of life upon the Sabbath any more than on the week-day. In short, opposite methods to those formerly employed for saving the souls, as well as the bodies, of men are now becoming familiar to us.

Medical text-books are quickly out of date, and he who would bring comfort and healing to the souls of men must likewise discover fresh and more refined modes of spiritual treatment. What is wrong in human nature is to be put right, not by compulsion and drastic measures, but by a quickening of the inner spiritual part of man. And if this analogy be any way correct, it would seem to follow that he who would minister to the wants of the body should not be—nay, cannot be—destitute of all religion. If man were but the curious compound of nerve and muscle that some depict him to be, then it might be sufficient for the doctor to take cognizance of nothing but the visible part. But, inasmuch as we believe that the flesh is in unison with the inner life that throbs beneath it, we require the same recognition in him who would approach us with a healing hand.

